# Supplementary figures and images for: Driving the blue fleet: Temporal variability and drivers behind bluebottle (Physalia physalis) beachings off Sydney, Australia
Source: PLoS One. 2022 Mar 17;17(3):e0265593. doi: 10.1371/journal.pone.0265593 (PMC8929625; doi:10.1371/journal.pone.0265593)

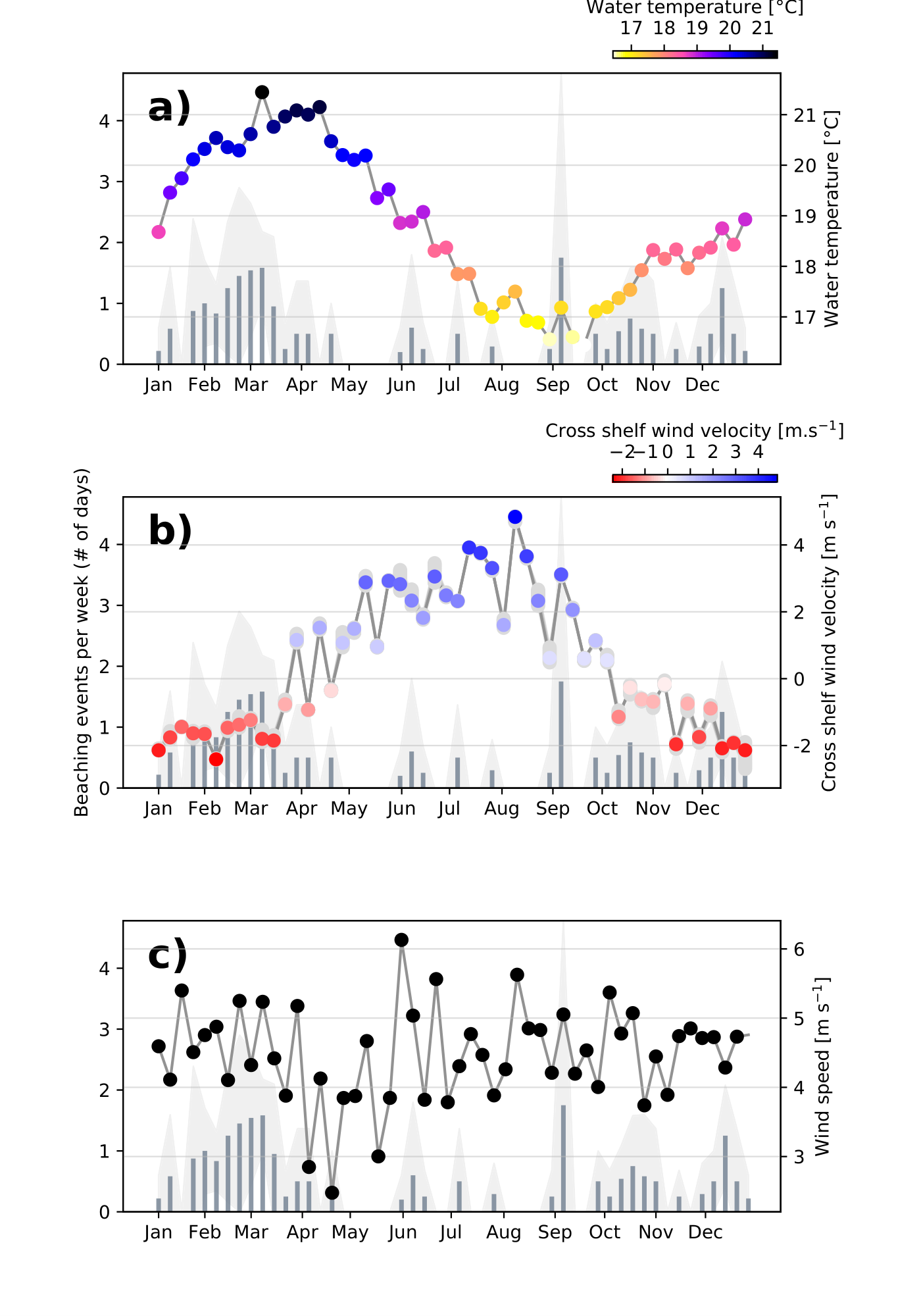

Supplement: S1 Fig — Grey bars on all panels show the number of beaching events per week over 2016-2020 and the standard deviation is shown in light grey shading. In panel a, the weekly mean water temperature is overlaid (right axis and colours). In panel b, the weekly mean cross-shore wind velocity component is overlaid (right axis and colours) with positive (negative) values showing wind from (towards) the coast. In panel c, then mean weekly wind speed is overlaid (right axis). (TIFF) [file pone.0265593.s001.tiff]
