# Supplementary material for: Driving the blue fleet: Temporal variability and drivers behind bluebottle (Physalia physalis) beachings off Sydney, Australia
Source: PLoS One. 2022 Mar 17;17(3):e0265593. doi: 10.1371/journal.pone.0265593 (PMC8929625; doi:10.1371/journal.pone.0265593)
Supplement: Attachment — Submitted filename: PONE-D-21-06875_R1_Editor_Revisions.pdf [file pone.0265593.s005.pdf]

Manuscript

Click here to access/download;Manuscript;natacha_bluebottles_template_R1.

Driving the blue fleet: Temporal variability and drivers behind bluebottle (*Physalia physalis*) beachings off Sydney, Australia

Natacha Bourg[1,¤a], Amandine Schaeffer[1,2], Paulina Cetina-Heredia[1,¤b], Jaz Lawes[3,4], Daniel Lee[1]

1 Coastal and Regional Oceanography Laboratory, School of Mathematics and Statistics, University of New South Wales, Sydney, New South Wales, Australia
2 Centre for Marine Science and Innovation, University of New South Wales, Sydney, New South Wales, Australia
3 Surf Life Saving Australia, Sydney, New South Wales, Australia
4 School of Biological Earth and Environmental Sciences, University of New South Wales, Sydney, New South Wales, Australia
¤a Mediterranean Institute of Oceanography, Université de Toulon, Aix-Marseille Univ, CNRS, IRD, Toulon, France
¤b College of Natural and Computational Sciences - Department of Natural Science, Hawai'i Pacific University, Honolulu, USA
* natacha.bourg@univ-tln.fr

Abstract

Physalia physalis, the bluebottle in Australia, are colonial siphonophores that live at the surface of the ocean, mainly in tropical and subtropical waters. The tentacles of *P. physalis* can deliver intense stings and these organisms can be present in large swarms, therefore having an impact on public health and coastal commercial and fisheries activities. *P. physalis*, who does not swim, is advected by ocean currents and winds acting on its gas-filled sail. While previous studies have attempted to model the drift of *P. physalis*, little is known about its region of origin, distribution, and the timing of its arrival to shore. In this study, we present a dataset with four years of daily *P. physalis* beaching and sting reports at three locations off Sydney's coast in Australia. We investigate the spatial and temporal variability of *P. physalis* presence in relation to different environmental parameters. This dataset shows a clear seasonal pattern where *P. physalis* beachings occur ~~more~~ in the Austral summer and are less likely in winter. Cold ocean temperatures do not hinder the presence of *P. physalis* and the two seasonal cycles are out of phase by 3-4 months. We identify wind direction as the major driver of the temporal variability of *P. physalis* arrival to the shore, both at daily and seasonal time-scales. The differences observed between sites and the occurrence of beaching events is explained by the geomorphology of the coastline which influences the frequency and direction of favorable wind conditions. This study is a first step into understanding the dynamics of *P. physalis* transport and ~~to~~ ultimately be able to predict its arrival to the coast and mitigate ~~the number of people who experience~~ painful stings ~~and require medical help~~.

Introduction

Physalia physalis is commonly known by beach-goers as the Portuguese Man-Of-War in the Atlantic Ocean, and as the bluebottle jellyfish in the Indian and Pacific Ocean (also

called the Indo-Pacific Portuguese Man-of-War). It is a colonial Cnidarian, part of the Order Siphonophorae, comprising interdependent, highly modified zooids that rely on each other to survive (Totton and Mackie, 1960). *P. physalis* is globally distributed, though is predominantly found in tropical and subtropical waters (Munro et al., 2019). These pelagic organisms are effective predators using their stinging cells to paralyze and feed on fish and fish larvae (Munro et al., 2019; Purcell, 1984). *P. physalis* can be present in large swarms and since their tentacles deliver intense stings, this can have impacts on public health and coastal commercial and fisheries activities (Munro et al., 2019). *P. physalis* stings are rarely lethal (only a few deaths recorded, e.g. (Burnett JW, 1989)), but can cause lifelong scarring and systemic symptoms such as gastrointestinal, muscular, cardiac, neurological and allergic reactions (Cegolon et al., 2013; Prieto et al., 2015). In Australia, many marine stings are treated by surf lifesaving personnel (surf lifesavers and lifeguards) who, between 2009/10 and 2019/20, have treated on average 40,128 stings each year, placing pressure on surf lifesaving service delivery and resources (SLSA, 2020).

Community and economic impacts of *P. physalis* presence along the shore and *P. physalis* morphology are relatively well understood (Cegolon et al., 2013; Munro et al., 2019), yet our understanding of drivers that affect *P. physalis* distribution, seasonality, abundance and transport to the coast is limited. Off the eastern coast of Australia, stranding of *P. physalis* typically occur more in summer. This is consistent with the suggestion that colonies mostly reproduce in autumn, and have a lifecycle of approximately 12 months (Ferrer and Gonzalez, 2020). *P. physalis* cannot actively swim; thus, their distribution is uniquely driven by physical and environmental atmospheric and oceanographic conditions in conjunction with their specific biological traits. One of the dominant zooids in each *P. physalis* is the pneumatophore, which is filled with gas and enables the juveniles and mature specimens to float on the surface of the ocean, hence subject to ocean currents and waves. The pneumatophore is bilaterally flattened and acts as a sail, directly subject to the wind forces, which is assumed to be the main driving force of *P. physalis* movements. It is tilted to the right or to the left (Totton and Mackie, 1960), causing an individual to drift at a certain angle to the wind direction. This sail can be moderately contracted and erected by an individual *P. physalis* and is, along with the elongation and contraction of its tentacles, the only active influence a *P. physalis* has on its transport (Munro et al., 2019). To date there has been no evidence to demonstrate whether (and to what extent) these controls can alter *P. physalis*' course (Iosilevskii and Weihs, 2009), and the movement and distribution of *P. physalis* is usually attributed to winds, currents and waves.

Previous studies have linked *P. physalis* beach stranding to environmental conditions and model their arrival to the coast, but have usually focused on unusual events (e.g. swarms)~~.~~ Ferrer and Pastor (2017); Prieto et al. (2015) studied massive beaching events that occurred in summer 2010 off the Basque coast (Spain) and the Mediterranean Basin using lagrangian particle tracking. Ferrer and Pastor (2017) proposed that offshore origin of these beached *P. physalis* was strongly dependent on the wind parametrisation used in the lagrangian tracking model. They therefore concluded these beaches *P. physalis*, were likely to have originated from the northern part of the North Atlantic Subtropical Gyre, thousands of kilometers away. They proposed wind as a dominant driver of *P. physalis* transport both off and on the coast~~.~~ Prieto et al. (2015) suggested that the massive arrival of *P. physalis* to the coast had been strongly influenced by zonal winds. Since then, massive beachings of *P. physalis* off the coast of Ireland in autumn 2016 (August and October) prompted further research (Headlam, 2020) to identify source populations ~~of *P. physalis*.~~ Results suggested that the population of *P. physalis* may have originated from the North Atlantic Current, supporting the findings of Ferrer and Pastor (2017). Using sting reports from five

summers across eight locations in New Zealand, Pontin et al. (2009) developed a neural network-based model to simulate the arrival of *P. physalis* towards the shore while assessing the contribution of large-scale winds and waves. They show that wave direction far from the shore can transport swarms to the studied region, while wind direction and speed one day before sting reports, in cells close to the shore strongly influence the strandings of *P. physalis*. A more recent and similar study by Pontin et al. (2011) extended the analysis to different regions all around New-Zealand. They validate results found by Pontin et al. (2009) that both wind and wave influence the occurrence of *P. physalis* strandings, while highlighting that different oceanographic regimes driving the beachings occur in different study areas. Unlike previous studies, a recent survey from Canepa et al. (2020) recorded nearly continuous strandings of *P. physalis* in Chile for three years, with the highest densities in the winter seasons two years in a row. These massive events coincided with an El Niño Southern Oscillation (ENSO) perturbation, with warmer ocean temperature conditions, and positive zonal wind anomalies (westerlies) transporting *P. physalis* to the coast, further highlighting wind as the major driver of *P. physalis* [illegible]ements and variability.

In this study, we present the temporal variability of *P. physalis* beaching and sting reports over three locations off Sydney, Australia. We investigate the link between the presence of *P. physalis* on the shore and environmental variables, including local winds, ocean temperatures, waves, and ocean currents, focusing on the daily and seasonal variability. Finally, the chances of *P. physalis* standings are related to typical wind sectors, and discussed in lights of the coastline orientation. The paper intends to build our understanding of environmental drivers leading to *P. physalis* strandings at three popular Australian beaches that could also inform coastal safety practice and mitigate sting risks to the community.

Data Methods

Study area

Fig 1. Map of the study area off eastern Australia showing the location of the 3 different beaches (Clovelly, Coogee and Maroubra). The location of Kurnell meteorological station is also shown (KN). The Windrose in the top left shows the daily wind distribution measured at KN from 2016 to 2020 and the four wind sectors used in this study, which are aligned with the local coastline. Top right : Satellite image of the different beaches (From The Gateway to Astronaut Photography of Earth). Bottom right : picture of a beached *P. physalis*.

Our study area is located on the southeast coast of Australia, and encompasses three beaches off Sydney that extend over $\approx$ 5.5 km of coastline. From May 2016 to May 2020, the occurrences of *P. physalis* beachings were reported daily by the lifeguards of three different Sydney locations : Clovelly (151.25^{o}E, 33.91^{o}S), Coogee (151.25^{o}E, 33.92^{o}S), and Maroubra (151.25^{o}E, 33.95^{o}S) (Fig 1 ; Fig 2). Maroubra is the most exposed and the longest beach (980m), and it is oriented directly towards the east. Coogee is smaller (410m) and more southward oriented. Note that Coogee has a small rocky outcrop (known as the Wedding Cake Island) 740m from the beach, which limits wave action on the beach. Clovelly beach is more south-oriented and is at the end of a narrow bay, hence more protected than the two other beaches (Fig 1).

Fig 2. Distribution in time of the water temperature reported from 2016 to 2020 (Blue circles) for a) Clovelly, b) Coogee, c) Maroubra. Days when beachings have been reported are shown by grey bars. Days when no beaching report is available (NaN) are scattered in black and shown at the top.

P. physalis datasets

We present two datasets that record the presence of *P. physalis*; beachings and stings. Beachings are recorded daily by the council lifeguards, written around 9AM for each beach, and are qualitative desciptions of P. physalis presence on the beach: "None", "Likely", "Some" or "Many". The dataset runs 4 years, from May 2016 to May 2020 (Fig 2). For this paper, we considered "Likely" days to be non-beaching days, and combined "Some"and "Many" to be observed. The resulting beaching dataset is then binary : 0 for absence, 1 for presence of *P. physalis.* At Coogee and Maroubra (Fig 2 b ; Fig 2 c), daily observational reports cover May 2016 and May 2020 with 94% and 93% data coverage (e.g. observations are missing on some public holidays when different life guards are on duty). In contrast, beaching reports at Clovelly beach only cover the warmer season, from October to April since the beach is not patrolled every day in winter (Fig 2 a).

In addition, we explore the variability of surf lifesaver sting reports for the same three sites. This reports list the number of people stung by *P. physalis* and treated by the surf lifesavers between 2016 and 2020, during the weekends and public holidays during patrol season (September - April). Estimates on beach attendance was also available from 2018 to 2020. For example, on a weekend day in summer, Coogee records more than four thousand people on the beach and a maximum of 350 stings.We explored whether sting numbers were dependent on numbers of beachgoers, but found no clear correlation between the two. Therefore, for these analyses we focus only on days when stings were recorded (rather than the daily presence/absence dataset), although it should be noted that days when no stings were recorded does not equate to no *P. physalis* in the water. To reduce the influence of outliers, we only consider stings when 10 or more were recorded a day, which occurred 6%, 9% and 10% of all patrolled days for Clovelly, Coogee and Maroubra, respectively. Comparing the beaching and sting datasets for matching days and locations (although different authors), only 7.9%, 15.8%, and 32.3% of the stings corresponded to a day when beaching was also at Clovelly, Coogee, and Maroubra, respectively. The daily match between these two datasets needs more investigation (see Discussion) and longer time-series. In the following, we focus on the beaching reports for the temporal variability, and we look at composite conditions during beach and stings.

Environmental datasets

To determine the influence of environmental parameters on the transport of *P. physalis* to the shore, we investigate the link between beaching variability and other environmental variables which are known for their seasonality and/or importance on *P. physalis* transport: wind speed and direction, local ocean currents, water temperature, wave height and rip currents. Water temperature, rip currents and wave height data are estimated daily by the lifeguards. Wave height is described by six categories ranging from flat to very high wave height ($<$ 0.5 meter, 0.5 meter, 1 meter, 1.5 meters, 2 meters, $>$ 2 meters). Surface water temperature data is recorded with no decimal (Fig 2). Rip currents estimates are qualitatively described by the lifeguards as "minimal", "be cautious", or "dangerous". We use wind measurements from the Kurnell weather station (ID: 66043), as recommended by E. Wood et al. (2012) as a proxy for

offshore winds. This station is located 8, 11 and 12 kilometers away from Maroubra, Coogee and Clovelly beaches respectively (Fig 1). Wind data is recorded every half an hour. The wind zonal and meridional components were averaged over 24 hours prior to *P. physalis* observations, in agreement with results from a lag correlation analysis (Fig. 3) and previous methods (Pontin et al., 2009). The wind is daily averaged 24 hours before 9AM local time when looking at beaching reports, and before 5PM local time for the sting reports. Predominant winds in this area are north-easterly, westerly and southerly, as shown on the windrose in Fig 1. For further detail on the monthly variability of winds, we refer to (Wood et al., 2016).

Local ocean currents time-series were also considered. We analyse ocean current velocity data from close-by moorings along the coast. One mooring is located above the 100 m isobath 2 km from the shore, and another above the 65 m isobath 10 km from the shore (SYD100 and ORS065, respectively, described in Roughan et al. (2013); Schaeffer et al. (2013)). The mooring's instruments measure U (zonal) and V (meridional) current velocity components throughout the water column every 5 minutes and 4-8 meters in depth. Here, we used daily averages at the shallowest bins (11 m and 12 m, respectively). The coastal oceanic circulation in this region is mostly driven by the East Australian Current (EAC) and its eddies (Roughan et al., 2019). The EAC is a strong western boundary current flowing southward along the coast of southeastern Australia, closing the western boundary of the South Pacific subtropical gyre. The EAC usually separates from the coast around 32^oS (Cetina-Heredia et al., 2014), a couple of hundreds of kilometers North of our study area. Downstream, off Sydney, mesoscale anticyclonic eddies are persistent (Oke et al., 2019), leading to a predominantly southward ocean circulation off Sydney with currents transporting tropical water, including on the continental shelf (Ribbat et al., 2020; Schaeffer et al., 2013).

Statistical analysis

Fig 3. Pearson correlation coefficient between beaching events at Maroubra and different environmental variables, for different lags. A negative lag means considering variables a day before the beaching day.

To quantify the importance of the different environmental variables on the coastal presence of *P. physalis*, taking into account the strong auto-correlation of the time-series, we used a Generalised Estimating Equations (GEE) (Liang and Zeger, 1986) model with an autoregressive AR(1) structure. The response was the binary beaching event variable at Coogee and Maroubra from 2016 to 2020, and the predictors were the wind zonal (cross-shore) and meridional (along-shore) components, the water temperature, wave heights, rip currents estimates and the ocean currents zonal and meridional components, as described in previous section, as well as a variable accounting for any seasonal effect defined as : $\sin(\frac{2\pi day}{365})+\cos(\frac{2\pi day}{365})$. We report Wald tests using a naïve variance estimator. When considering daily time-series over the four years, the wind variable is considered lagged by 1 day, while other variables are averaged over the same day, based on the maximum value of the lagged cross-correlation Pearson coefficient between beaching events and wind components (Fig. 3). Both the GEE analysis and correlation analysis were performed from daily time-series as explained above (qualitative beaching), and also from weekly time-series, using the number of beaching events per week from 2016 to 2020 as a response variables (quantitative), and weekly averaged values of environmental data as predictors, and the variable accounting for any seasonal effect defined as : $\sin(\frac{2\pi week}{365})+\cos(\frac{2\pi week}{365})$.

Results

Temporal variability of *P. physalis* beaching

Between 2016 and 2020, daily observations of beachings from the lifeguards show that the occurrence of *P. physalis* off Sydney varies both temporally and spatially (Fig 2 ; Fig 4), with the beaching frequency differing from one beach to another. Maroubra is where *P. physalis* is the most likely to be sighted, with 132 beaching events over four years, followed by Coogee with a total of 82, and Clovelly with 38 beaching days (summer only). These differences can be related to differences in the beach lengths and exposures to open ocean. In descending order Maroubra is the largest, followed by Coogee, while Clovelly is far narrower than the others. In addition, the Wedding Cake Island located in front of Coogee, and the enclosed geography of Clovelly, may prevent the arrival of *P. physalis* (Fig 1). Simultaneous beaching in Maroubra and Coogee occurs only 10-20% of the beaching days, and the correlation between the timeseries of beaching presence at the daily timescale is $r = 0.1$, increasing to $r = 0.3$ when considering the weekly number of beachings.

For Coogee and Maroubra where daily data is available all year long, beaching events display a strong seasonal signal, with frequent events in the Austral summer (DJF), and very unlikely events in the Austral winter (JJA) (Fig 2 ; Fig 4). Indeed, between 2016 and 2020, 50% and 46% of strandings occurred during the three months of summer in Maroubra and Coogee respectively. In Maroubra, spring is (after summer) the second season with most beaching events (30% of beachings), whereas in Coogee, beaching events are more numerous in autumn (25%) than spring. Interestingly, there are still instances of winter beaching for Coogee and Maroubra, up to 10% of annual sightings in Coogee. This suggests that despite the seasonal cycle to their strandings, *P. physalis* survive winter time and cold temperatures and can still be advected to the coast. Therefore, the seasonality of their strandings is likely influenced by environmental parameters rather than only driven by their lifecycle.

Fig 4. Bar plot for each beach, showing the occurrence of observed beaching events for each season (see legend) from the 2016-2020 daily lifeguard data. The total occurrences indicate the number of days over the 4 years and the percentages for each season are relative to the total numbers of occurrences per beach (e.g. in Maroubra 50% of the beachings occurred in summer). Only summer months are shown for Clovelly as it is the only full season.

Drivers of *P. physalis* transport to shore

The link between environmental variables and the spatial and temporal variations in *P. physalis* arrival to the shore is first investigated at the daily timescale. Table 1 shows the output of the GEE analysis using beaching at Maroubra or Coogee as the response variable. This analysis revealed a significant relationship with the wind time-series. In particular, a common dependence on cross-shore wind ($p < 0.05$) was observed for both Coogee and Maroubra, suggesting that wind directed both towards and away from the coast is the main driver for beachings, while along-shore wind is not related to beaching events. The negative coefficient for cross-shore wind shows that negative zonal winds (i.e. towards the shore) are likely to lead to a beaching event according to the statistical model.

Wave height, water temperature, rip currents and ocean currents, do not appear to be significant drivers of beachings. This is consistent with beaching events that have occurred at both cold (16 ^{o}C) and warm (23 ^{o}C) water temperatures (Fig 2), hence

Table 1. Outputs of GEE analysis on daily data. Response variable is the binary beaching event at Maroubra (left cells) and Coogee (right cells). Significant values are marked in red.

Predictors	Coefficient	95% CI	p-value
Wind U velocity	-0.23 — -0.31	(-0.44;-0.02) — (-0.60;-0.02)	0.03 — 0.04
Wind V velocity	-0.03 — 0.12	(-0.16;0.11) — (-0.06;0.29)	0.69 — 0.19
Water temperature	-0.04 — 0.08	(-0.38;0.29) — (-0.36;0.53)	0.80 — 0.72
Wave height	0.38 — 0.04	(-0.24;1.00) — (-0.89;0.96)	0.23 — 0.94
RIP currents	0.36 — 0.39	(-0.33;1.06) — (-0.29;1.07)	0.30 — 0.26
Water U velocity	0.84 — -3.33	(-4.38;6.06) — (-9.63;2.96)	0.75 — 0.30
Water V velocity	0.12 — -0.91	(-1.62;1.87) — (-3.05;1.23)	0.89 — 0.10
Seasonality	0.47 — 0.58	(-0.30;1.23) — (-0.64;1.79)	0.23 — 0.35

water temperature does not prevent nor significantly drive beachings. Although ocean currents are thought to play a role in *P. physalis* transport and regions of origin (Ferrer and Pastor, 2017; Headlam, 2020), we find no clear pattern between ocean velocity measured by Acoustic Doppler Current Profiler (ADCP) and observed beaching events. Note that this could be due to the dataset, since the shallowest bin of the ADCP used here is located at a 11m depth, while the main body of *P. physalis* colonies usually only reach few centimeters. Data of currents at the ocean-atmosphere interface would then be necessary to further investigate this correlation.

Fig 5. Summer rose plots showing wind conditions of days before days with more than 10 stings (Stings) reported, wind conditions of days prior *P. physalis* beachings (Beaching) were observed, and prior to days when no beachings of *P. physalis* were observed (No Beaching) for each beach. Each beach is shown with the yellow line.

Since wind stress appears to be a dominant driver, we investigate the composite wind conditions for beachings and stings. Focusing on summer, when the majority of sightings occur, rose plots of wind conditions for 24 hours preceding P. physalis sightings show that north-easterly and south-easterly (i.e. shoreward winds) are the two most favourable directions P. physalis (Fig 5), while no beaching occurs from southwesterly winds. There are spatial differences between the locations : north-east is the most favourable wind condition for beaching at Coogee and Maroubra, while it is south-east for Clovelly. Sting reports and beaching show similar favourable conditions for P. physalis in Maroubra and Coogee, but a few differences in Clovelly (Fig 5). At Clovelly, beachings are reported during north-easterly and southerly winds (consistent with the beach orientation), but few stings are reported during the latter. This discrepancy may be due to weather conditions : north-easterly winds usually occur on sunny days, while southerly winds are often grey and rainy, influencing the number of days with beach-goers and their exposure to stings. Besides, north-easterly winds usually occurs in the afternoon at these locations when beach attendance is high and stings more likely, while beachings are recorded in the mornings by lifeguards.

Seasonality of environmental variables

Since *P. physalis* is more common in summer (See Section: Temporal variability of *P. physalis* beaching), but not impossible in winter (hence still alive), the question is whether environmental variables drives its seasonality. Fig 6 provides a view of seasonal cycles of beaching events at Maroubra for each week of the year (averaged over the four years), together with cross-shore winds, wind speed, and water temperature. Although the water temperature displays a strong seasonal signal (Fig 6 a), it does not have the

Table 2. Outputs of GEE analysis on weekly averaged data. Response variable is the number of days with beaching event per week at Maroubra (left cells) and Coogee (right cells). Significant values are marked in red.

Predictors	Coefficient	95% CI	p-value
Wind U velocity	-0.03 — -0.01	(-0.04;-0.01) — (-0.02;0.00)	0.00 — 0.06
Wind V velocity	-0.01 — 0.01	(-0.02;0.01) — (0.00;0.03)	0.33 — 0.03
Water temperature	-0.00 — 0.00	(-0.03;0.03) — (-0.02;0.03)	0.90 — 0.75
Wave height	0.02 — 0.02	(-0.05;0.10) — (-0.05;0.09)	0.51 — 0.62
RIP currents	0.01 — -0.04	(-0.09;0.11) — (-0.10;0.03)	0.84 — 0.29
Water U velocity	-0.03 — 0.05	(-0.59;0.53) — (-0.43;0.52)	0.92 — 0.85
Water V velocity	0.03 — -0.05	(-0.11;0.17) — (-0.16;0.07)	0.67 — 0.43
Seasonality	0.02 — 0.01	(-0.04;0.08) — (-0.04;0.06)	0.53 — 0.64

Fig 6. Weekly climatology of beaching events and environmental variables at Maroubra. Grey bars on all panels show the number of beaching events per week over 2016-2020 and the standard deviation is shown in light grey shading. In panel a, the weekly mean water temperature is overlaid (right axis and colours). In panel b, the weekly mean cross-shelf wind velocity component is overlaid (right axis and colours) with positive (negative) values showing wind from (towards) the coast. In panel c, then mean weekly wind speed is overlaid (right axis).

same phase as the seasonal signal of beachings (shown by the grey bars) which peak in early February, while ocean temperatures are maximum in late March. Nevertheless, the correlation between the seasonality of beachings and ocean temperature becomes more significant when a lag is added, with a maximum correlation coefficient ($r = 0.27$, $p = 4.10^{-4}$) found for a lag of 15 weeks (i.e. between 3 and 4 months). This suggests that the seasonality of temperature could have a delayed effect, such as on *P. physalis* growth 3-4 months before being advected to Sydney.

Consistent with previous results (See Section: Drivers of *P. physalis* transport to shore), the seasonality of wind direction visually matches the annual cycle in *P. physalis* beaching. In particular, while the wind speed shows no seasonal cycle comparable to the beaching variability (Fig 6 c), the weekly mean of the cross-shelf component of the wind shows negative values, hence a wind blowing towards the shore (easterlies) in the first and last 12 weeks of the year, when sightings of *P. physalis* are frequent. Conversely, positive values, hence a wind blowing predominantly from land (westerlies) is dominant in winter when *P. physalis* rarely reach the coast (Fig 6 b). The maximum sightings also occur during the strongest easterly wind (weeks 6 and 52) and no beaching occurred during the strongest westerlies (weeks 28-30, 32-33).

This visual link between seasonality of beachings and cross-shore winds is confirmed by the GEE analysis, performed over 4 years but on a weekly timescale. The p-values of the model (Table 2) show that for the 2 sites, cross-shore wind is highly significant for beaching (Table 1). Interestingly, results for Coogee also show significant p-values for meridional components of the wind velocity. This difference could be explained by the fact that Coogee is more oriented towards the South than Maroubra. This important difference could explain the discrepancy and low correlation coefficient between the timeseries of beaching events at Maroubra and Coogee, and their differences in seasonality (Fig 4).

Chances of *P. physalis* per beach and wind sector

Since wind direction appears to be the main driver for *P. physalis* beaching at both the daily and the weekly time scales, we want to investigate how likely it is to see *P. physalis* during favourable conditions and how it varies between seasons and sites. For each beach, we quantify the percentage of instances when each wind direction brought *P. physalis* to the shore (Table 3 ; Table 4). Overall, for any wind condition, Maroubra is where beachings are more likely, followed by Coogee and Clovelly (Table 3). Again, these differences are explained by the morphology of each beach shown on Fig 1. For example, favourable wind conditions for *P. physalis* beaching in Maroubra and Coogee (i.e. North-East followed by South, Fig 5) are more frequent than those for beaching in Clovelly (i.e. South followed by North-East), explaining the differences in *P. physalis* abundance between the locations (Fig 1). At Maroubra, most of the beaching events are associated with north-easterly and south-easterly winds the day prior to sightings, with similar chances of beaching (i.e. 16-17% respectively, Table 3). Focusing on summer only, the north-easterly seabreeze is more likely to lead to a beaching event (Fig 5), with a 24% chance of *P. physalis* beaching (Table 4). The chance of beachings during westerly winds (North-West and South-West) are negligible, showing that winds from the coast usually prevent the arrival of *P. physalis* to shore. South-East and North-East are also the favourable wind directions for beaching at Coogee (with 10-11% and 12-13% chances respectively in summer). The difference between North-East and South-East is smaller at Coogee than at Maroubra (Table 3 ; Fig 5); this difference could be explained by the orientation of Coogee, which is oriented more towards the South than Maroubra. Winds favourable for beachings at Clovelly are different from the two other beaches, with southerly winds being the most favourable condition (13% of chance of beaching), followed by north-easterly winds (Fig 5 ; Table 3). It should be noted that for any wind condition, beachings are more likely to occur in summer (Table 3), suggesting that the seasonality of beaching events is not only driven by winds. This point is further addressed in Discussion.

Table 3. Frequency of beaching events per wind sector.

Chances of beaching when	Clovelly		Coogee		Maroubra	
NE	X	4 %	10%	11%	17%	24%
SE	X	12 %	12%	13%	16%	22%
SW	X	6 %	2%	5%	2%	0%
NW	X	0 %	1%	0%	1%	0%

Blue: computed on data 24 hours before a sighting, all year round from 2016-2020, black: on summer dates only from 2016-2020.

Table 4. Frequency of stings per wind sector.

Chances of stings when	Clovelly	Coogee	Maroubra
NE	20 %	33%	40%
SE	29 %	22%	16%
SW	0 %	5%	5%
NW	0 %	0%	0%

Computed data 24 hours before a report, on summer dates only from 2016 to 2020.

The chance of stings displayed in Table 4 show more frequent stings than beachings for any wind condition. This could be due to the differing datasets, but also to the fact

that only beached *P. physalis* are reported by lifeguards, while stings can occur over a larger area (on the beach and in adjacent water) and during the whole day. Despite these differences in abundance, the wind conditions transporting *P. physalis* to the shore are qualitatively the same. Indeed, as with the beachings, it is unlikely for stings to be reported after westerly winds for all three locations. For Coogee and Maroubra, North-East is the most favourable condition followed by South-East, while it is the opposite for Clovelly. Interestingly, north-easterly and south-easterly winds have almost equal chances to be followed by beachings, although the chance of stings is more than two times higher during north-easterly than south-easterly wind conditions. Similar patterns were also observed for Coogee, but to a lesser extent. As already mentioned, there is an association between sting reports and the presence of people recreating in the water which can be influenced by the weather conditions, with north-easterly winds often associated with warm sunny days.

Discussion

This study is the first one to explore *P. physalis* beaching observations in relation to various environmental variables in Australia. Using multiple datasets collected from three proximate locations off Sydney's coast, our results show that the occurrence of beachings differ in time as well as from one beach to another. We also demonstrate a strong relationship between this spatio-temporal variability and wind direction at the daily timescale, with North-East and South clearly identified as favourable wind directions for *P. physalis* beachings at these locations. The differences in occurrence of observed beaching events among close-by beaches are likely ~~to be~~ explained by the geomorphology of the coastline as well as by the differences in frequency of favourable wind conditions. Besides, the year-round dataset over four years enables the identification of a clear seasonal pattern in the frequency of beaching events at both Coogee and Maroubra. Most *P. physalis* beachings occurred in austral summer (DJF), with the least number of beachings recorded in winter (JJA). Analyses comparing the time variability of beaching events and different environmental variables show that winds towards the shore are ~~clearly~~ associated with beaching's seasonality; hence, wind direction is proposed as a major driver of seasonal patterns observed in *P. physalis* beaching.

The relationship between wind, surface ocean currents, and the motion of *P. physalis* was first investigated by Woodcock (1944). They studied *P. physalis* drifting direction with wind and observed a clear tendency of *P. physalis* moving at around 45° of the wind direction. The drifting angle of *P. physalis* and its asymmetry was later extensively studied by Totton and Mackie (1960) using field observations and conducting experiments. They found that left (right) handed *P. physalis* drifted at 40° to the right (left) to the wind direction under light winds (under < 8 m s^{-1}), and drifted in the direction of the wind under stronger winds. These concepts were extended in 2009 by Iosilevskii and Weihs (2009), when a theory regarding *P. physalis* transport was developed by comparing its hydrodynamics to a wind-powered sailboat. This study shows the complexity of *P. physalis* hydrodynamic relationship with winds and surface currents. *P. physalis* drifting angle to the wind is now believed to be approximately 40° and is suggested to vary with wind speed and with the size of *P. physalis* (Iosilevskii and Weihs, 2009; Totton and Mackie, 1960). Due to the lack of data on *P. physalis*'s handedness, this has not been investigated in the present study, but a suggested theory is that wind influence *P. physalis* arrival in nearshore waters such that one direction (e.g. North-East) will push left-handed *P. physalis* to the coast while the other (e.g. south) will more likely push the right-handed individuals. It is important to highlight that the wind is a major contributor to the ageostrophic component of the surface current

(influencing circulation and generating local waves), and stokes drift and wind-induced currents are known to be highly relevant in regard to the transport of passive tracers, especially in the first centimeters of the ocean. Then, this relationship between beaching events and cross-shore wind can be explained by the wind drag on *P. physalis* outside of water, but also by the action of wind-induced current on its transport.

When analysing the beaching and sting datasets, some of the findings were unexpected.

There are days where *P. physalis* beachings were sighted while no stings were reported and vice-versa. As the number of stings depends on the number of people present in the water, no stings reported does not necessarily mean that no *P. physalis* were present in the water. Even if sting and beaching reports do not match on a daily basis, the results regarding their link to environmental variables are quite similar across both datasets. We also identified North-East and South as typical wind conditions when stings occurred, and the differences between beaches and wind directions were similar to results using the beaching reports. Differences between the two datasets could be explained by the difference in the timing of the reports but also by the nature of the reports (stings happen in the water, while beachings are reported only when *P. physalis* are stranded on the shore).

Some beaching events were recorded during winter months that are dominated by westerly winds, for example 10 % of beaching events off Coogee occurred in winter (Fig 4). In addition, there was a high frequency of beaching events in spring recorded during weeks that were dominated by south-westerly winds, as can be observed during September and May in Fig 6. This results are surprising since beachings would not be expected when wind is coming from land, if wind were the only driving variable. Moreover, we found that beachings are more likely in summer under any wind conditions (Table 3). This suggests that wind is not the sole driver of *P. physalis* beachings and that other physiological or environmental variables such as sea state and ocean currents could also influence *P. physalis* transport and subsequent beaching events. Stokes drift, the movement caused by wave propagation, can also have an important role on the drift of organisms and inert particles in the ocean (i.e. plastic (Onink et al., 2019); (Feng et al., 2011)), and is likely relevant to *P. physalis* transport. However, our attempt to link ocean current and wave height with *P. physalis* beachings was not conclusive. This may be due to the type of current observations used in the analyses being too deep (the shallowest measure was at 11 m). Observations of ocean currents closer to the surface and of higher resolution may be necessary to expose any dependence of beaching events on these variables.

To date, little is known about the ecology, lifecycle and pathways of *P. physalis*. It has been suggested that colonies have a lifecycle of approximately 12 months (Ferrer and Gonzalez, 2020) but specific details about the duration are lacking. Environmental factors such as light, temperature, salinity and food availability may have an effect on jellyfish reproduction and growth rates, though it is not yet known if there are seasonal influences on *P. physalis* (Ferrer and Gonzalez, 2020; Munro et al., 2019; Purcell et al., 2012). The results presented in this study demonstrate that *P. physalis* can be present close to the coast year-round and show that abundances may fluctuate during the year but also from a year to another. We do not find ocean temperature (at least within the range reached at these latitudes) to be determining on beaching events, with cold water not preventing the presence of *P. physalis* (e.g. beachings observed on the 01/09/2019 with water at 16°C). However, the seasonal cycle of ocean temperature at Sydney lagged by 3-4 months (similar to temperature at lower latitudes) is shown to be correlated with beaching events. We can therefore not exclude the possible influence of sea surface temperature on *P. physalis* lifecycle. Besides, our results show that local winds are important in nearshore waters, but we also hypothesise that ocean circulation

offshore may be important in transporting *P. physalis*, for example from tropical zones to the temperate latitudes (where our observations are from), with the EAC being the main pathway. This also supports that more beachings occur over summer as the EAC separates further South from the continent in summer months than in winter (Oke et al., 2019). Still, the unknown source location and variability in offshore abundance of *P. physalis* is a major source of uncertainty in this study and a clear knowledge gap to be addressed in future research.

Another limitation is derived from the observational dataset provided by council lifeguards, where the reliability of counts assessing *P. physalis* beaching may be affected by human subjectivity. To reduce this in future studies we propose future data collection to include more detailed records including *P. physalis* size and morphology (left or right-handed). Records of an estimated number of *P. physalis* beached, as well as sustained observations at more locations would also be beneficial.

To conclude, our four year observational database of *P. physalis* beachings off Sydney, showed a clear seasonal signal of beachings in this area, with most beaching events occurring in summer. Comparing the seasonal signal to different environmental variables, we identified a strong dependence of beaching events with wind direction at seasonal and ~~also at~~ daily timescales. These results are in agreement with literature that suggested that the wind plays an important role in *P. physalis* transport in other study areas (e.g. (Canepa et al., 2020; Ferrer and Pastor, 2017; Pontin et al., 2009; Prieto et al., 2015)). We expect these results to be valid for *P. physalis* arrival to the coast worldwide. However, the role of other variables need to be further investigated when more data is available, in particular for unexpected and extreme beaching events.

Supporting information

S1 Fig. Weekly climatology of beaching events and environmental variables at Coogee. Grey bars on all panels show the number of beaching events per week over 2016-2020 and the standard deviation is shown in light grey shading. In panel a, the weekly mean water temperature is overlaid (right axis and colours). In panel b, the weekly mean cross-shelf wind velocity component is overlaid (right axis and colours) with positive (negative) values showing wind from (towards) the coast. In panel c, then mean weekly wind speed is overlaid (right axis).

Acknowledgments

The authors would like to thank Duncan Rennie, Manager Public Safety and Aquatic Services, as well as the Randwick City Council and all lifeguards and surf lifesavers who collected the datasets. Wind measurement dataset was obtained from the Bureau of Meteorology (`http://www.bom.gov.au/places/nsw/MV07/observations/kurnell/`). Mooring data was sourced from Australia's Integrated Marine Observing System (IMOS) – IMOS is enabled by the National Collaborative Research Infrastructure strategy (NCRIS). It is operated by a consortium of institutions as an unincorporated joint venture, with the University of Tasmania as Lead Agent. IMOS data is freely accessible at `https://portal.aodn.org.au/`. The authors would also like to thank Stats Central from UNSW for their guidance with novel statistical analyses and the anonymous reviewers for their invaluable feedback during the preparation of this manuscript for publication.

References

Burnett JW, G. W. (1989). A fatal jellyfish envenomation by the portuguese man-o'war. *Toxicon.*

Canepa, A., Purcell, J., Cordova, P., Fernandez, M., and Palma, S. (2020). Massive strandings of pleustonic portuguese man-of-war (physalia physalis) related to enso events along the southeastern pacific ocean. *Latin American Journal of Aquatic Research*, 48:2020.

Cegolon, L., Heymann, W., Lange, J., and Mastrangelo, G. (2013). Jellyfish stings and their management: A review. *Marine drugs*, 11:523–50.

Cetina-Heredia, P., Roughan, M., Sebille, E., and Coleman, M. (2014). Long-term trends in the east australian current separation latitude and eddy driven transport. *Journal of Geophysical Research: Oceans*, 119.

Feng, M., Caputi, N., Penn, J., Slawinski, D., de Lestang, S., Weller, E., and Pearce, A. (2011). Ocean circulation, stokes drift, and connectivity of western rock lobster (panulirus cygnus) population. *Canadian Journal of Fisheries and Aquatic Sciences*, 68(7):1182–1196.

Ferrer, L. and Gonzalez, M. (2020). Relationship between dimorphism and drift in the portuguese man-of-war. *Continental Shelf Research.*

Ferrer, L. and Pastor, A. (2017). The portuguese man-of-war: Gone with the wind. *Regional Studies in Marine Science*, 14:53 – 62.

Haywood, L. (2010). Killer jellyfish strikes in italy. *The Sun.* Accessed on 05/03/2011.

Headlam, Jasmine L.; Lyons, K. K. J. L. E. S. Q. D. T. G. H. W. D. M. M. D. T. K. A. (2020). Insights on the origin and drift trajectories of portuguese man of war (physalia physalis) over the celtic sea shelf area. *Estuarine, Coastal and Shelf Science.*

Iosilevskii, G. and Weihs, D. (2009). Hydrodynamics of sailing of the portuguese man-of-war physalia physalis. *Journal of The Royal Society Interface*, 6(36):613–626.

Liang, K.-Y. and Zeger, S. L. (1986). Longitudinal data analysis using generalized linear models. *Biometrika*, 73(1):13–22.

Munro, C., Vue, Z., Behringer, R., and Dunn, C. (2019). Morphology and development of the portuguese man of war, physalia physalis. *Scientific Reports*, 9.

Oke, P. R., Roughan, M., Cetina-Heredia, P., Pilo, G. S., Ridgway, K. R., Rykova, T., Archer, M. R., Coleman, R. C., Kerry, C. G., Rocha, C., Schaeffer, A., and Vitarelli, E. (2019). Revisiting the circulation of the east australian current: Its path, separation, and eddy field. *Progress in Oceanography*, 176:102139.

E. Wood, J., Roughan, M., and Tate, P. (2012). Finding a proxy for wind stress over the coastal ocean. *Marine and Freshwater Research*, 63:528.

Wood, J. E., Schaeffer, A., Roughan, M. and Tate, P. M. (2016). Seasonal variability in the continental shelf waters off southeastern Australia: Fact or fiction? *Continental Shelf Research*, 112, 92-103 doi:10.1016/j.csr.2015.11.006

Onink, V., Wichmann, D., Delandmeter, P., and van Sebille, E. (2019). The role of ekman currents, geostrophy, and stokes drift in the accumulation of floating microplastic. *Journal of Geophysical Research: Oceans*, 124(3):1474–1490.

Pontin, D., Schliebs, S., Worner, S., and Watts, M. (2011). Determining factors that influence the dispersal of a pelagic species: A comparison between artificial neural networks and evolutionary algorithms. *Ecological Modelling*, 222(10):1657–1665.

Pontin, D., Worner, S., and Watts, M. (2009). Using time lagged input data to improve prediction of stinging jellyfish occurrence at new zealand beaches by multi-layer perceptrons. volume 5506, pages 909–916.

Prieto, L., Macías, D., Peliz, A., and Ruiz, J. (2015). Portuguese man-of-war (physalia physalis) in the mediterranean: A permanent invasion or a casual appearance? *Scientific Reports*, 5.

Purcell, J. (1984). Predation on fish larvae by physalia physalis, the portuguese man of war. *Marine Ecology Progress Series*, 19.

Purcell, J., Fuentes, V., Atienza, D., Olariaga, A., Talves, U., Colahan, C., and Gili, J.-M. (2012). Temperature effects on asexual reproduction rates of scyphozoan polyps from the nw mediterranean sea. *Hydrobiologia*.

Ribbat, N., Roughan, M., Powell, B., Rao, S., and Kerry, C. (2020). Transport variability over the hawkesbury shelf (31.5Äì34.5¬∞s) driven by the east australian current. *PLOS ONE*, 15:e0241622.

Roughan, M., Schaeffer, A., and Cresswell, G. (2019). East australian currentòÜ. In Cochran, J. K., Bokuniewicz, H. J., and Yager, P. L., editors, *Encyclopedia of Ocean Sciences (Third Edition)*, pages 340 – 350. Academic Press, Oxford, third edition edition.

Roughan, M., Schaeffer, A., and Kioroglou, S. (2013). Assessing the design of the nsw-imos moored observation array from 2008-2013: Recommendations for the future. In *OCEANS 2013 MTS/IEEE - San Diego: An Ocean in Common*, pages 1–7, United States. Institute of Electrical and Electronics Engineers (IEEE).

Schaeffer, A., Roughan, M., and Morris, B. (2013). Cross-shelf dynamics in a western boundary current regime: Implications for upwelling. *Journal of Physical Oceanography*, 43:1042–1059.

Surf Life Saving Australia, Sydney. (2020). National coastal safety report 2020.

Totton, A. and Mackie, G. (1960). *Studies on Physalia Physalis (L.): Pt.1. Natural History and Morphology ; Pt.2. Behaviour and Histology.* Discovery reports. Cambridge University Press.

Woodcock, A. H. (1944). A theory of surface water motion deduced from the wind-induced motion of the physalia. *Journal of Marine Research*, 5:196.

Click here to access/download;Figure;Fig2.tiff

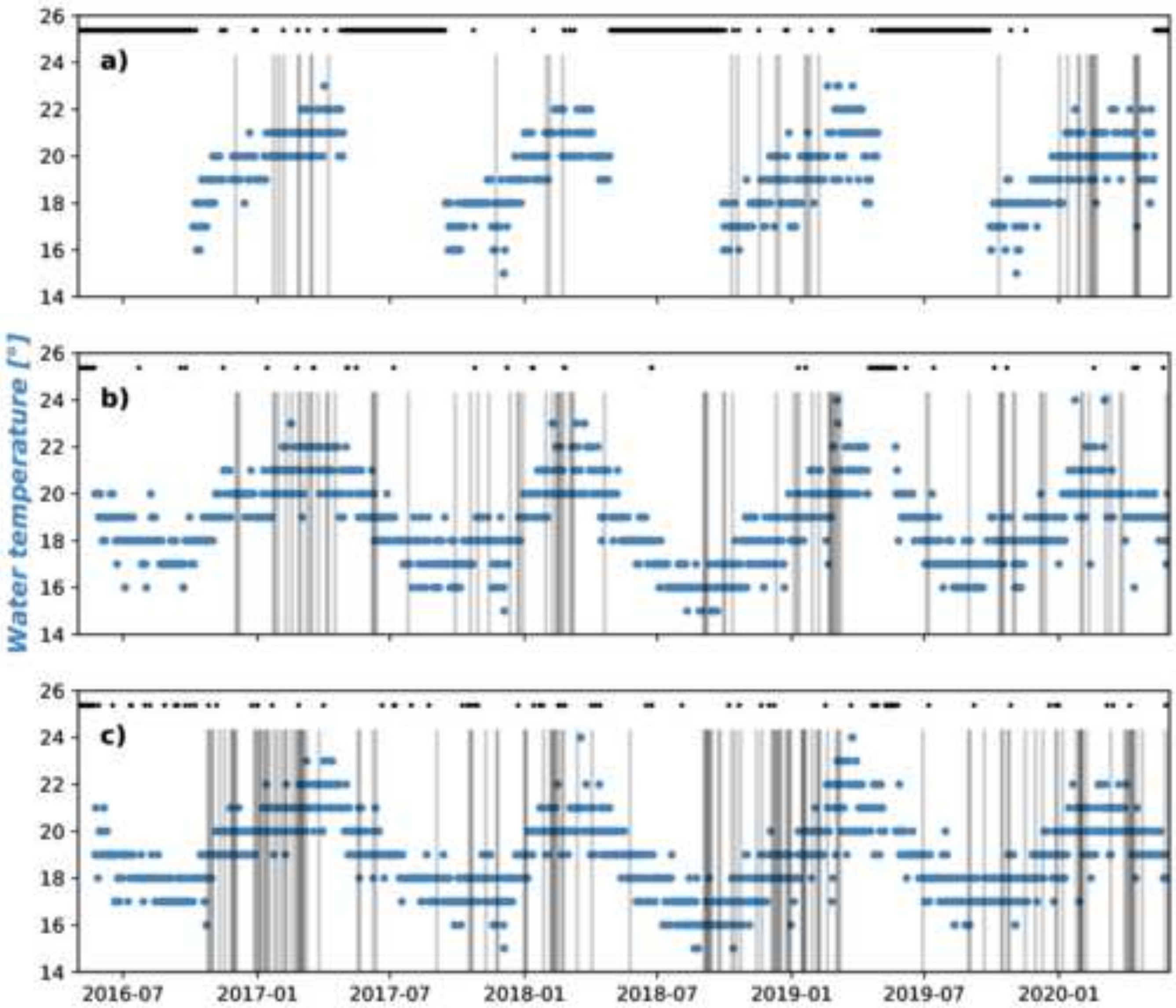

Click here to access/download;Figure;Fig3.tiff

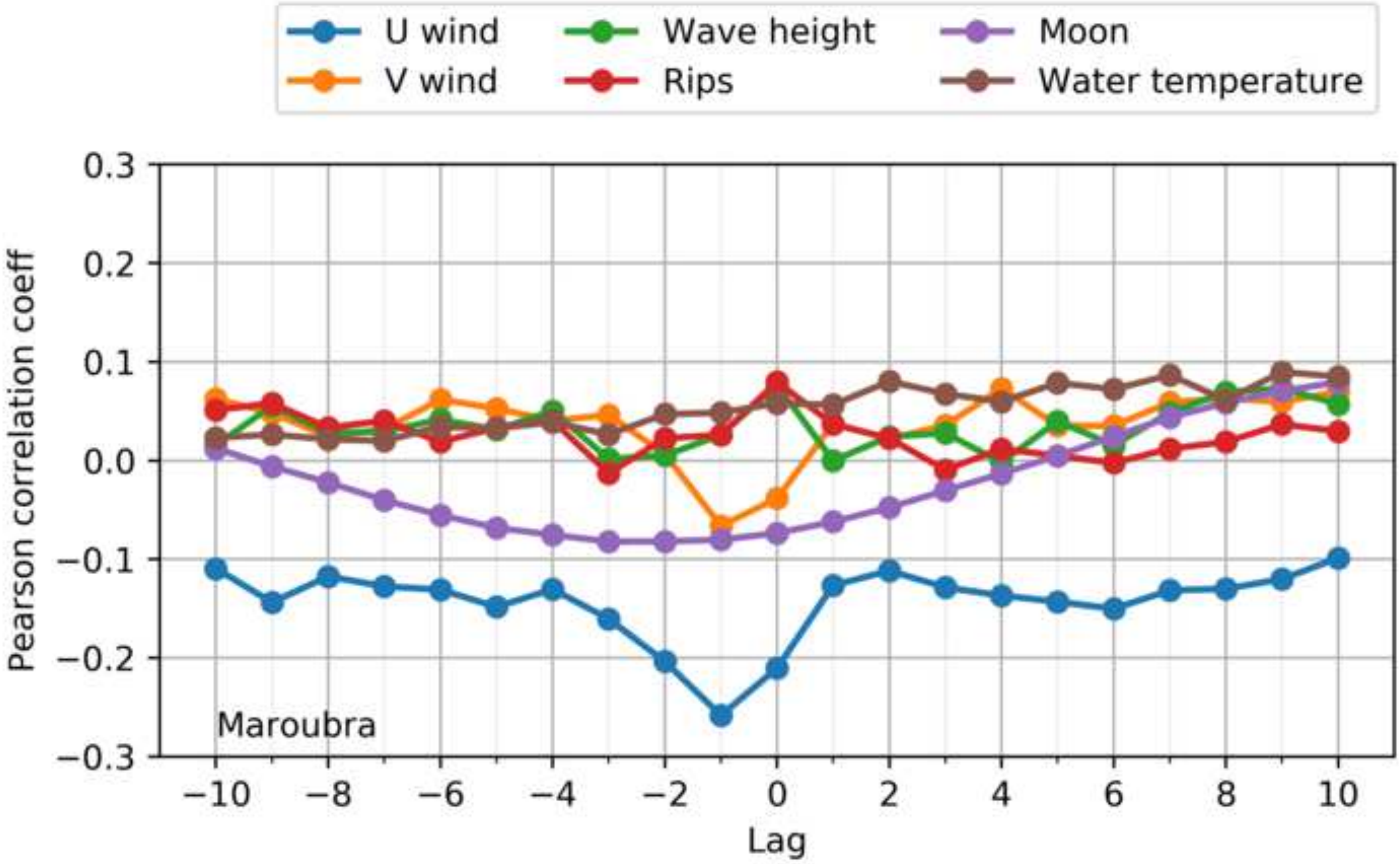

Click here to access/download;Figure;Fig4.tiff

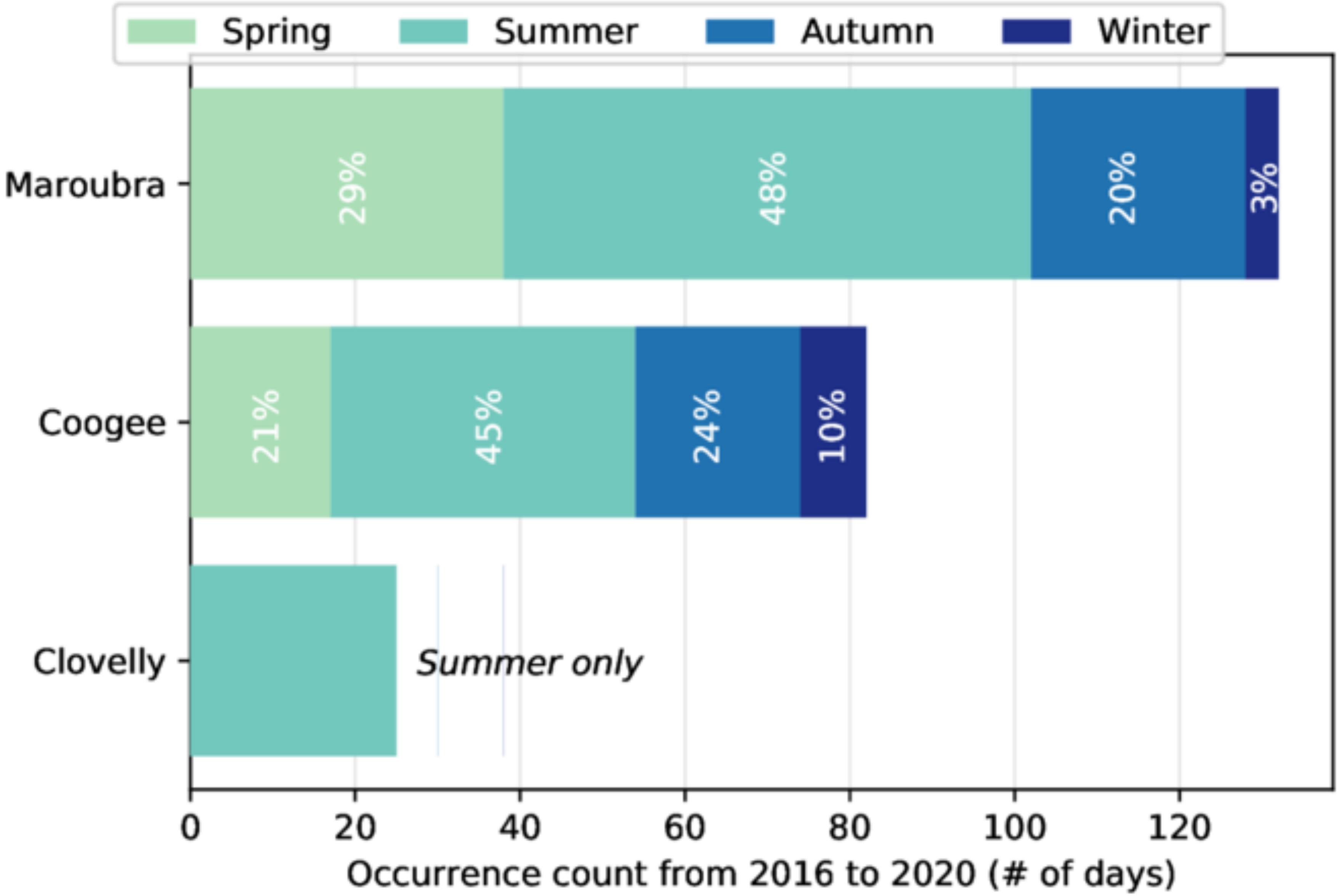

Click here to access/download;Figure;Fig5.tiff

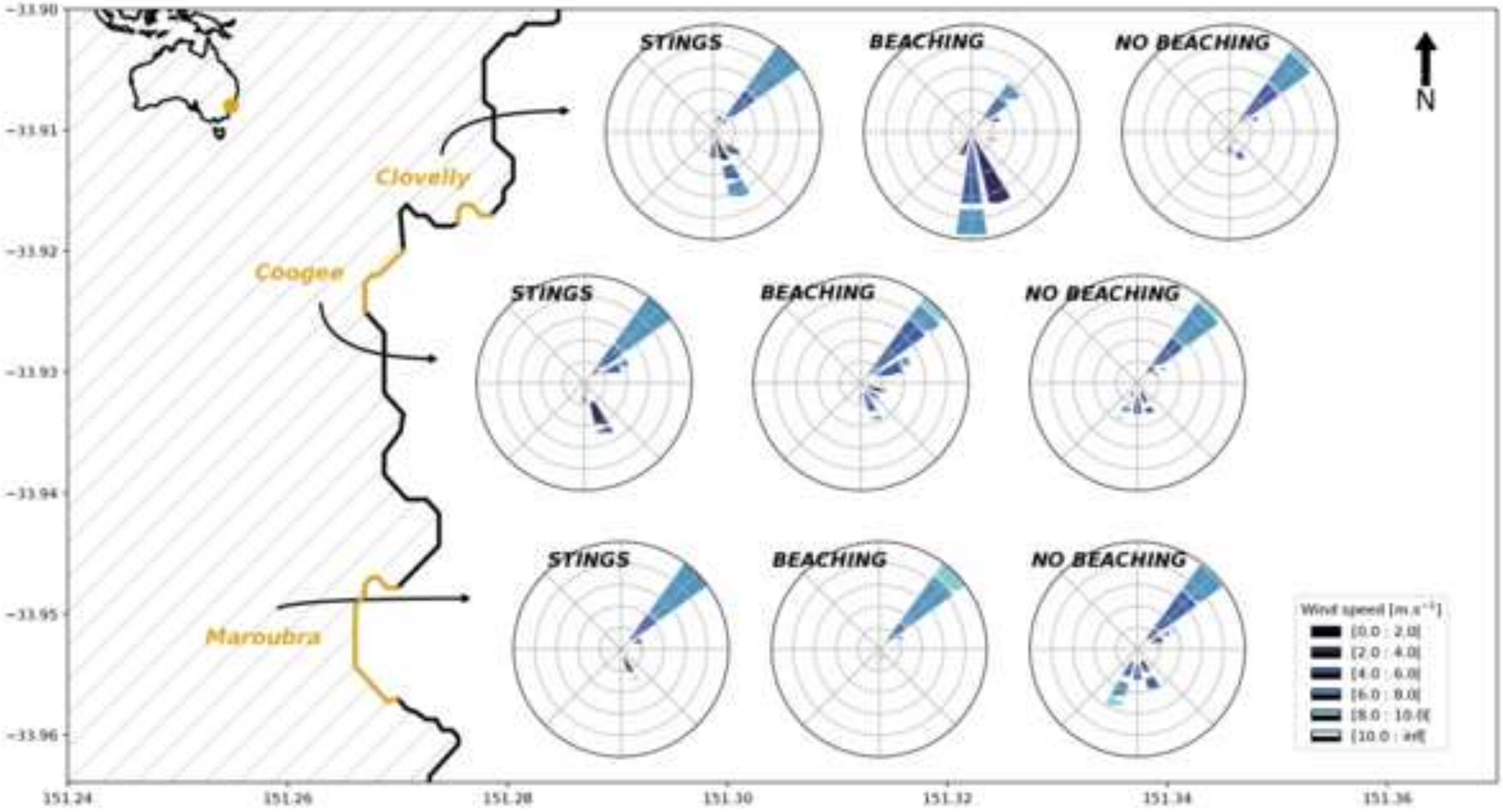

Click here to access/download;Figure;Fig6.tiff

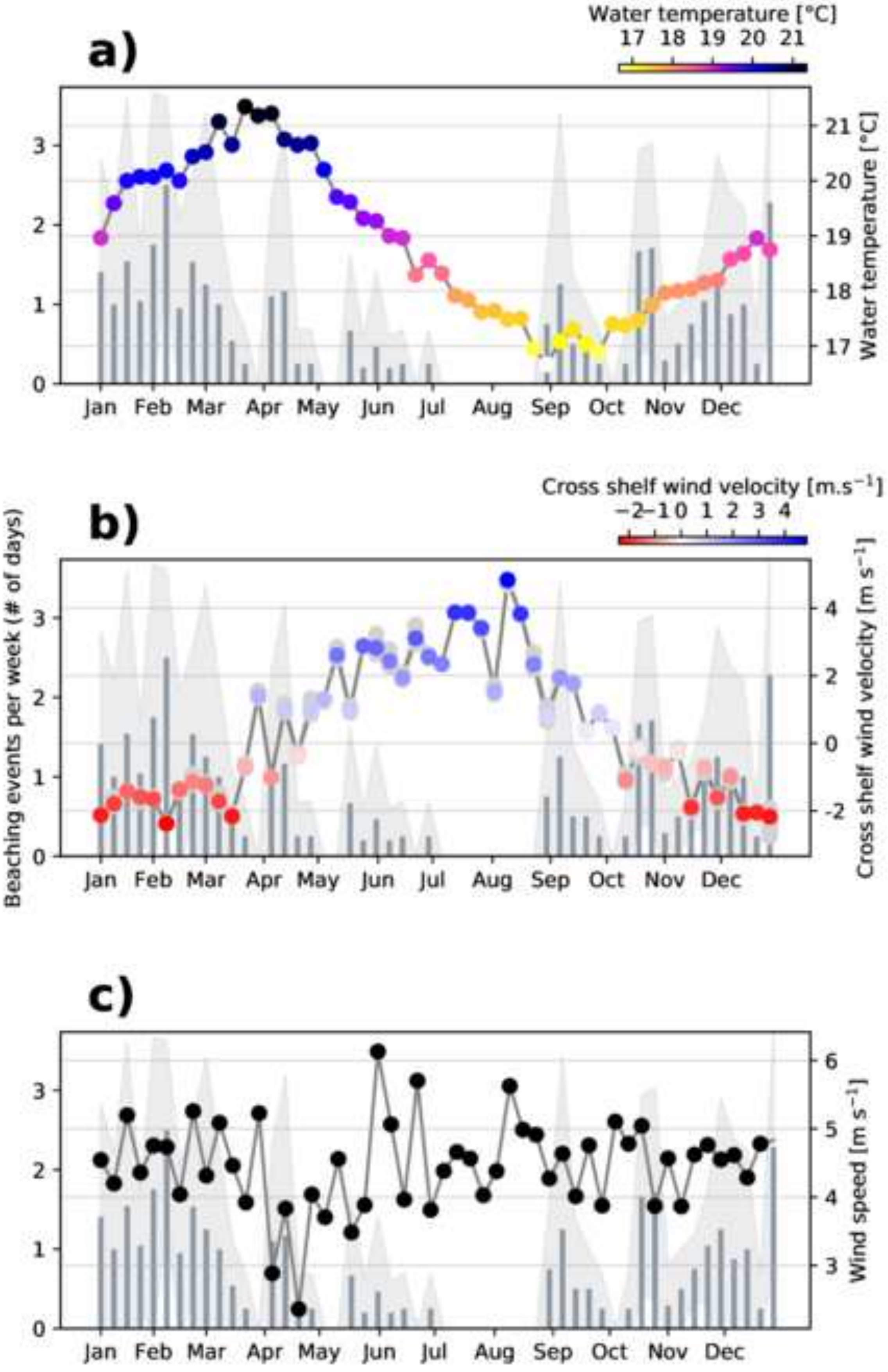

Figure

Click here to access/download;Figure;Fig1.tiff

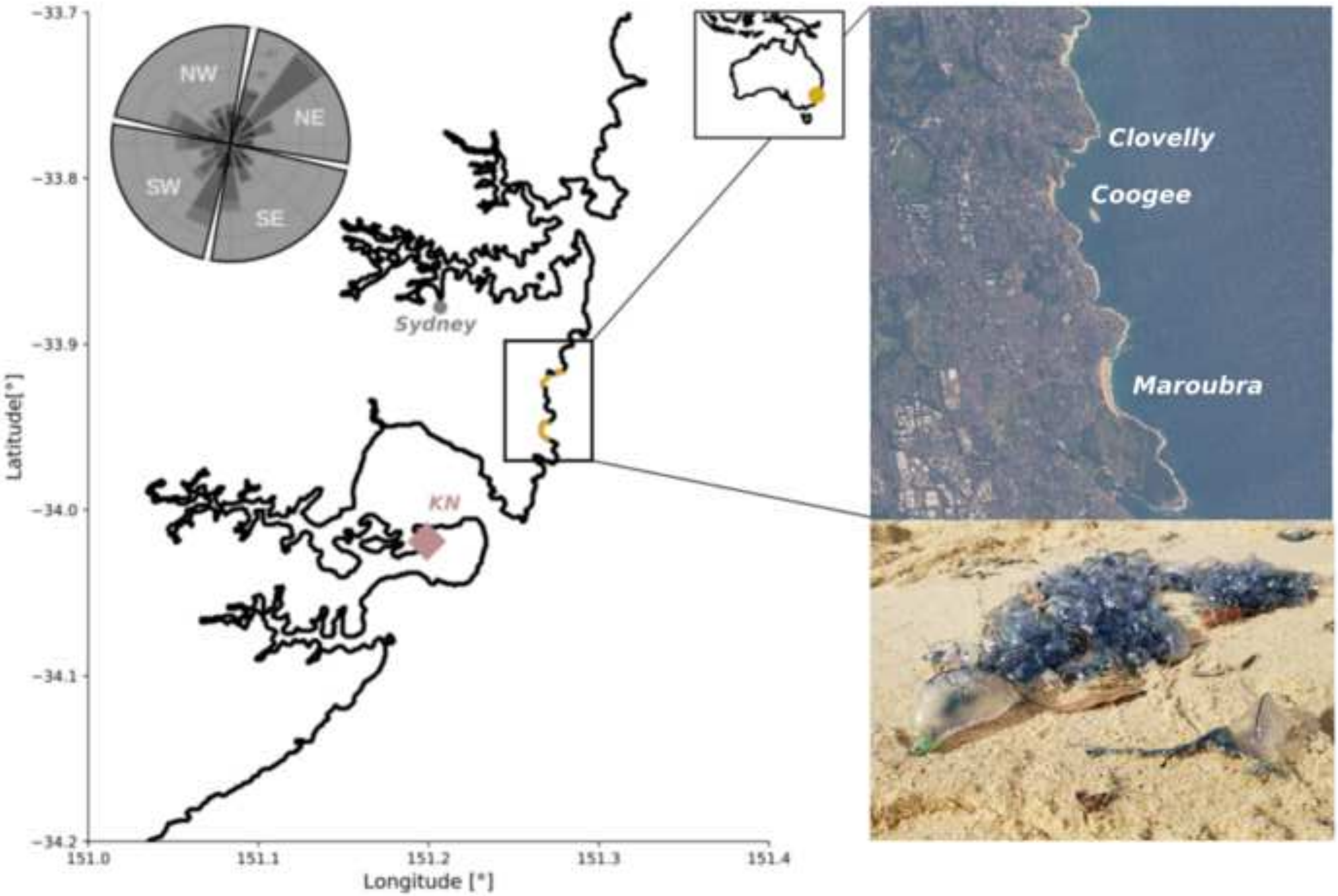

Click here to access/download
Supporting Information
S1_Fig.tiff

Click here to access/download
Supporting Information
Supporting_Information_Physalia_Dataset.xlsx
