# Supplementary material for: Driving the blue fleet: Temporal variability and drivers behind bluebottle (Physalia physalis) beachings off Sydney, Australia
Source: PLoS One. 2022 Mar 17;17(3):e0265593. doi: 10.1371/journal.pone.0265593 (PMC8929625; doi:10.1371/journal.pone.0265593)
Supplement: Attachment — Submitted filename: Review_response.pdf [file pone.0265593.s006.pdf]

Response to the comments about the submitted paper *PONE-D-21-06875R1*

Dear Editor,

We would like to thank you for the opportunity to revise and improve the manuscript.

Please find below a brief description of the major changes in the manuscript, which we made to address the main concerns that came up in the revision. All the detailed comments have also been modified with the revised manuscript. We hope that this revised version will be suitable for publication in PLoSONE.

We look forward to hearing from you,

Natacha Bourg and co-authors.

Answer to specific comments

Comment 1 *The paper describes analyses, but does not provide the necessary details to interpret the results Line 111: "We explored whether sting numbers were dependent on numbers of beachgoers, but found no clear correlation between the two" Can you please explain how this was done and what were the results?*

Answer to 1 We have now expanded the analysis to specifically investigate the link between stings and beach attendance. Since the variables are strongly skewed, we used the step-wise GEE analysis, adding beach attendance as a predictor for the sting presence/absence timeseries. This analysis highlights that the beach attendance is a significant predictor (p = 0.001), but not the most important one. Please see Table 2 line 242 in the manuscript.

The scatter plot of Fig R1 is included below to confirm the skills of the model. It presents the probability predicted by the model (x-axis) versus real sting events (y-axis) data; the colour is the density of points. A logistic-like behaviour can be seen for the threshold probability around 0.3. This means that for probabilities $x \in [0; 0.3]$ the model generally predicts no beaching ($y = 0$), while for probabilities $x \in [0.3; 1]$ the model generally predicts a beaching event ($y = 1$).

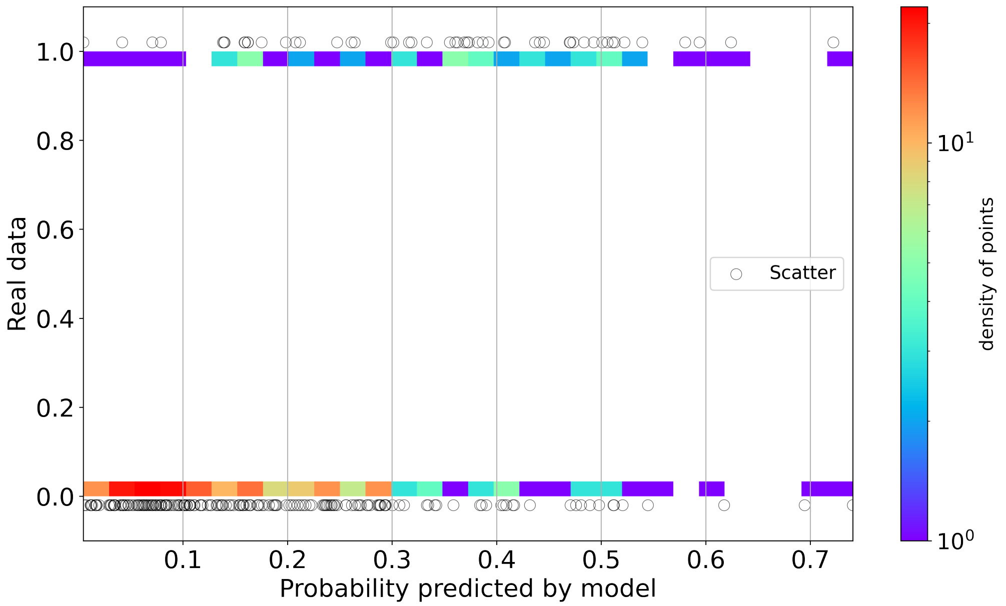

Figure R1: Scatter plot (black circles) of the prediction of the GEE model (x-axis) against the real sting data (y-axis). In colour is the density of points of the scatter.

Comment 2 *Line 121: "The daily match between these two datasets needs more investigation". These statements also need more detailed reporting of the analyses. It is not possible to assess what was done and what is the correspondence between "stings" and "beachings".*

Answer to 2 We have investigated how many days have simultaneous beaching and sting reports at all sites. The manuscript now reads lines 106-114: "It should be noted that days when no stings were recorded does not equate to no *P. physalis* in the water. To remove false negatives, we do not analyse the data on days with no beach attendance. For matching days and locations (although different authors), beaching and sting datasets do not daily compare and sting reports are more frequent than beaching reports. Only 8%, 16%, and 32% of the stings corresponded to a beaching day at Clovelly, Coogee, and Maroubra, respectively. Due to this mismatch, and to the lack of data

from April to September, the beaching dataset is the main material of the study and we use stings data to complete and nuance the analysis". We have also included all details for your interest in the table below (Fig. R2). You can see that the number of days when *P. Physalis* was present on the beach and stings were recorded is not high, which we discuss in the manuscript lines 328-335: "Differences between the two datasets could be explained by the difference in the timing of the reports but also by the nature of the reports (stings happen in the water, while beachings are reported only when *P. physalis* are stranded on the shore). The discrepancy may also be due to weather conditions : north-easterly winds usually occur on sunny days, while southerly winds are often grey and rainy, influencing the number of days with beach-goers and their exposure to stings. Also, north-easterly winds usually occur in the afternoon at these locations when beach attendance is high and stings more likely, while beachings are recorded in the mornings by lifeguards.".

**Clovelly**	**Lifeguard No Obs**	**Lifeguard Obs**	**No Data**	**Grand Total**
**Sting No Obs**	74	6	7	87
**Sting Obs**	35	3	4	42
**Grand Total**	**109**	**9**	**11**	**129**

**Coogee**	**Lifeguard No Obs**	**Lifeguard Obs**	**No Data**	**Grand Total**
**Sting No Obs**	87	9	2	98
**Sting Obs**	16	3		19
**Grand Total**	**103**	**12**	**2**	**117**

**Maroubra**	**Lifeguard No Obs**	**Lifeguard Obs**	**No Data**	**Grand Total**
**Sting No Obs**	76	6	11	93
**Sting Obs**	21	10	2	33
**Grand Total**	**97**	**16**	**13**	**126**

**Combined**	**Lifeguard No Obs**	**Lifeguard Obs**	**Grand Total**
**Sting No Obs**	53	13	66
**Sting Obs**	41	24	65
**Grand Total**	**94**	**37**	**131**

Figure R2: Summary of correspondence between sting and beaching reports for the three locations.

Comment 3 *There are several untested assumptions for the statistical tests. Line 169: NOTE: The binary response data are not normal - but binomial. The rip currents are rank data, not numerical.*

Answer to 3 Thank you for highlighting this. We have removed the parametric lag correlation analysis and corresponding Pearson correlation coefficients to properly include the rank data. This has been replaced by a new analysis shown in Fig 3 (line 143). Please see lines 144-151 in the manuscript : "We identify the temporal lag for which each variable is influencing the beaching of *P. physalis*, between λ = -7 to 0 days before the latter observations. Fig 3 shows the difference between the distribution of each variable when considering all data, or a subset when a beaching was recorded λ days later. We consider that the greater is the difference, the stronger is the relationship. The wind influence appears to be maximum for a lag of one day (Fig 3 a; Fig 3 b: the maximum of the red line is at λ=-1), while considering other variables the same day as

the beaching seems appropriate (Fig 3 c; Fig 3 d, Fig 3 e: the maximum of the red line is at λ=0)."

Comment 4 *Some of the discussion of the results compare proportions or discuss similarities, without actually performing the statistical analyses. For instance: Line 192 Did you attempt cross correlations between the two sites? This would be a much better way to assess covariability.*
Answer to 4 As beaching is a binary variable, we believe that displaying the percentage of simultaneous beachings between locations is a valuable and easily understandable comparison. Furthermore, we have added a correlation coefficient between stings timeseries off Maroubra and Coogee. See lines 187-190 in the manuscript. "Simultaneous beachings in Maroubra and Coogee occur only 14% of the beaching days. This number increases to 54% of simultaneous stings at the two beaches, and the correlation between the two timeseries of the number of stings is ($r = 0.3$, $p < 0.0001$)."

We also included a new method to investigate the similarity between the *P. Physalis* presence at the different sites. We have now joined the data of the two sites with year-round observations (Maroubra and Coogee) in a GEE analysis, with a variable "site" in the predictor variables. This way, we explore whether the site is important to model the presence of beachings and stings. Interesting new results show that the site is important for stings, but less so for beaching. Please see Tables 1 and 2 for the results in the manuscript

Note that we improved our statistical analysis, now running a backward step-wise GEE, in order to choose the best predictor variables from the available options. All details are shown and explained in sections "Statistical analysis" and "Drivers of *P. physalis* transport to shore", lines 153-157 and 262-242.

For your information, Fig R3 below shows the skills of the GEE model to predict the real beaching data (similarly to Fig R1 for stings). As stated in the manuscript, (line 155) we do not pretend to create a predictive model, but rather to highlight important drivers of the beaching. Nonetheless, considering the probability threshold around 0.1, the figure shows (to some extent) a logistic-like behaviour.

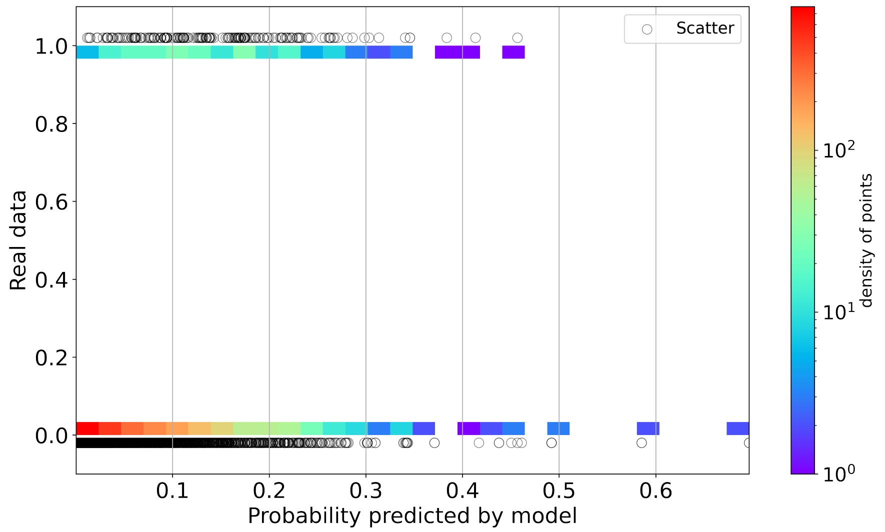

Figure R3: Scatter plot (black circles) of the prediction of the GEE model (x-axis) against the real beaching data (y-axis). In colour is a histogram showing the density of points of the scatter.

Comment 5 *Different beaches have different survey seasons and this complicates the interpretation.*
Answer to 5 The 3rd site (Clovelly) is not included in the statistical GEE model because it does not cover winter months, however Clovelly data remains relevant in regards to the beach orientation, which is radically different from the two other locations. Including all three sites in the other analyses therefore highlights the influence of the geomorphology of the beaches in *Physalia physalis* beaching abundance and favourable wind conditions (See Fig 6 and Tables 3 and 4 in the manuscript).

Comment 6 *Table 3 and Table 4: Can you please compare the observed proportions versus the expected proportions? How frequently are these wind conditions observed, will influence whether these results are significant or not.*
Answer to 6 This is a very good point indeed. We have therefore added to Tables 3 and 4 the number of instances of each wind sector in the data. Moreover, the new Figure 7 (see lines 264-289 for comments) now provides an overview of the wind direction distribution in function of the seasons, with the blue part of the windrose showing the beaching event proportion for each wind direction.

Comment 7 *Line 198: "Indeed, between 2016 and 2020, 50% and 46% of strandings occurred during the three months of summer in Maroubra and Coogee respectively. In Maroubra, spring is (after summer) the second season with most beaching events (30% of beachings), whereas in Coogee, beaching events are more numerous in autumn (25%) than spring" Can you please perform tests to determine if seasons matter: statistically speaking? Merely mentioning the proportions of events is not enough to determine whether these proportions are significantly different from what we would expect. Did you define the seasons equally, so each one accounts for 25% pf the time? This would be the expected proportions. But we need a way to assess if these proportions are significantly different from the observed proportions.*
Answer to 7 We would like to thank the editor for this good point that makes the results section considerably more robust. We have now added a variable accounting for seasonal effect in the GEE model. It is defined as:
seasonality = $\cos\left(2\,\pi \frac{dayofyear + maxbbday}{365}\right)$
where *dayofyear* is the day of the year (i.e. 31/12: 365, 01/01: 1... etc) and maxbbday is the day with the highest number of beachings on average over the four years (7th of February). The GEE analysis depicts *seasonality* as significant for beachings (see Table 1). In all our analyses the seasons are defined equally (including three months), with Summer: December January February, Autumn: March April May etc...

Comment 8 *The writing needs to be organized: much material needs to be moved: • From Methods to Introduction (see notes in the pdf) • From Results to Discussion (see notes in pdf)*
Answer to 8 Most of your suggestions on this have been helpful and followed, thank you. Please see the revised manuscript.

Comment 9 *The writing needs to be improved substantially to streamline the text, clarify the writing and fix some grammar and typos (like the persistent use of "data" in singular).*
Answer to 9 Thank you, we have re-checked and corrected the grammar and typos. Please see the revised manuscript.

Comment 10 *Finally, Page 3 – Figure Caption 1: Can you please credit the images. You used images from "Satellite image of the different beaches (From The Gateway to Astronaut Photography of Earth". Is this a free creative commons product? Can you provide a reference? I found the site, but there is no information on the use of these images: https://eol.jsc.nasa.gov/SearchPhotos/*
Answer to 10 We have added the proper references needed, please see Line 77 Caption of Figure 1: (Image courtesy of the Earth Science and Remote Sensing Unit, NASA Johnson Space Center, eol.jsc.nasa.gov , Picture ID :ISS037-E-20021).
