# Supplementary material for: Driving the blue fleet: Temporal variability and drivers behind bluebottle (Physalia physalis) beachings off Sydney, Australia
Source: PLoS One. 2022 Mar 17;17(3):e0265593. doi: 10.1371/journal.pone.0265593 (PMC8929625; doi:10.1371/journal.pone.0265593)
Supplement: Attachment — Submitted filename: PONE-D-21-06875_reviewer.doc [file pone.0265593.s003.doc]

SOME COMMENTS ABOUT THE PAPER:

After reading the article, I write down some recommendations and some thoughts that could help the authors to tweak some comments made throughout the article.

The authors use some apostrophes in grammatical structures where their use at the scientific article level may not be necessary or these structures can be rewritten. It is recommended to review its use with a native speaker, such as in:

…*P.physalis*'s morphology

…*P. physalis*'s course

Line 67:

For validation, these models were compared to massive beaching events that occurred in summer 2010 off the Basque coast (France) and the Mediterranean Basin.

The Basque coast is in Spain, in the autonomous community of the Basque Country. This community ends at the border between Spain and France. In the summer of 2010, the presence of *Physalia* occurred at several beaches along the coast. But on some important beaches, such as La Concha Beach (in the city of Donostia-San Sebastián), small fishing boats were transformed into cleaning boats and left the beach area to meet the Portuguese man-of-war and collect them before their arrival along the beach.

Line 194:

The few number of sightings in winter as well as P. physalis supposed lifecycle could be explained by a collective death in winter.

For me, a low or no number of winter sightings in the coastal area does not mean that there are a large number of deaths in the open sea. It is to be expected that there are always Portuguese man-of-war of different ages drifting for months in the great oceanic gyres (using the wind as main driver) with a peak of reproduction that could occur at the end of summer-beginning of autumn. On the European coast of the North Atlantic Ocean, it is typical that during the winter (not only in the months of summer) there is also a notable presence of small *Physalia* (3-5 cm long float, 3-4 months old) that due to the very strong southerly winds of successive storms (favourable to dragging towards the Bay of Biscay) have caused the appearance of *Physalia* on the coast to be anticipated. Therefore, the highest mortality possibly occurs when these organisms reach the dry beach, where they no longer leave and end up dying. These organisms do not appear to die from the severe winter conditions, at least in the North Atlantic Ocean. These conditions can make it possible for them to reach the coast at a time other than summer. It is for this reason that it is important to monitor the beaches outside of the time that the beaches are patrolled and during the lifetime of the organisms.

Line 348:

[11] similarly suggest that wind is a dominant driver of P. physalis transport, but propose wind to be more relevant offshore and ocean circulation becoming the main driver in nearshore areas.

[11] suggests that the wind is the most relevant mechanism both off and on the coast for this peculiar organism. The very superficial ocean circulation (considering this as the one that exists in the first 5 centimetres of the water column, where *Physalia* lives) in the great gyres of ocean circulation is greatly influenced by the wind, as shown by very low-weight drift buoys floating on the surface. The data of these buoys shows that the surface ocean circulation is far from following the Ekman theory (that is, generating a surface current at 45 degrees from the wind). It is for this reason that possibly the best solution to explain the drift of *Physalia* is to use the wind, because also the wind is the generator of local waves and the circulation at the upper centimetres of the water column.

Line 358:

In addition, there was a high frequency of beaching events in spring recorded during weeks that were dominated by south-westerly winds, as can be observed during September and May in Fig 5. This result is surprising since beachings would not be expected when wind is coming from land, if wind were the only driving variable.

To study the arrival of these organisms, it would be necessary to analyse not only the winds of the days prior to arrival, but also the evolution of winds throughout the life of these organisms, which could be from a few months to a year (more or less), depending on the size of the organism. Prevailing southwesterly winds could probably bring many *Physalia* located in the open sea below Australia. And winds from the northeast, east or southeast (in the days prior to arrival), even if they were of short duration, could cause these organisms to end up in the study beaches. Therefore, it is highly recommended to analyse the annual evolution of the wind in a very large area (several degrees in longitude and latitude) around the study area. Surely these organisms have been able to travel more than 10,000 kilometres on their journey to reach the beach.

Line 371:

Observations of ocean currents closer to the surface and of higher resolution (e.g. coastal High-Frequency RADAR) may be necessary to expose any dependence of beaching events on these variables.

The fundamental problem with using high-frequency radar observations to explain caravel drifts is that they provide information on currents at 1-3 meters above the surface. This information is quite different from that existing in the same ocean-atmosphere interface, that is, in the first centimeters of the water column. So to speak, the Portuguese caravel is a very light balloon (a caravel of 10 centimeters of float can weigh around 25 grams) that has tentacles that act as an anchor so that it does not fly. So it seems unlikely that trying to explain their drift with currents below 5-10 centimeters from the sea surface will not do much.
